# Malnutrition among the older adult: an additional challenge for the rehabilitation team-insights of a small population of Switzerland

**DOI:** 10.3389/fpubh.2024.1436566

**Published:** 2024-07-09

**Authors:** Bojan Miletic, Yves Sutter, Gordana Starčević-Klasan, Udo Courteney, Lejla Jelovica, Andrica Lekić, Silvije Šegulja

**Affiliations:** ^1^Department of Rehabilitation and Geriatrics, Lucerne Cantonal Hospital, Wolhusen, Switzerland; ^2^Department of Medical Sciences, Faculty of Health Studies, University of Rijeka, Rijeka, Croatia; ^3^Department of Internal Medicine, Lucerne Cantonal Hospital, Wolhusen, Switzerland

**Keywords:** geriatrics, malnutrition, Mini Nutritional Assessment, rehabilitation, treatment

## Abstract

**Introduction:**

Nutritional deficiency among the older adult is a widespread concern, significantly affecting their health. The prevalence of malnutrition increases with age, concurrent health conditions, and the level of care provided. Hospital stays can affect as 90% of the older adult. Malnutrition can hinder efforts to improve health and regain function in individuals undergoing rehabilitation. This study aims to assess the frequency of malnutrition among geriatric rehabilitation patients.

**Materials and methods:**

A retrospective quantitative analysis was conducted on 357 patients admitted to the geriatric unit at Lucerne Cantonal Hospital Wolhusen in Switzerland. The patients’ nutritional status was evaluated using the standardized Mini Nutritional Assessment Long Form questionnaire.

**Results:**

The initial analysis indicated a considerable prevalence of malnutrition among the geriatric population: 31.1% were identified as malnourished (MNA < 17), while an additional 35.8% were at risk of malnutrition (MNA 17–23.5), totaling 66.9% of patients. The Kruskal-Wallis ANOVA test revealed a statistically significant difference in MNA scores among different age groups (*p* = 0.035). Statistical analysis also suggested a slightly higher prevalence of malnutrition among female patients. The duration of rehabilitation varied from 20.07 ± 6.93 to 22.20 ± 7.50 days, with longer durations associated with lower MNA scores. A correlation analysis between MNA values and body mass index (BMI) showed a positive correlation coefficient (*r* = 0.56), indicating that lower MNA scores were associated with lower BMI and vice versa.

**Conclusion:**

Malnutrition is prevalent among individuals over 65 years old, highlighting the importance of regular and timely nutritional assessments for geriatric patients to mitigate the complications and enhance prognoses in both acute care and rehabilitation settings. Such assessments can also improve the efficacy of rehabilitation programs and potentially reduce the duration of rehabilitation, thus carrying significant economic implications.

## Introduction

Malnutrition in geriatrics is a prevalent and concerning issue that significantly impacts the health and well-being of older adults. The World Health Organization has highlighted the importance of healthy aging in its work on aging between 2016 and 2030, emphasizing the need for efforts across sectors (1). Globally, malnutrition affects around 23 to 46% of older adults (2). The prevalence of malnutrition increases with age, existing health conditions, and the quality of care in hospital settings where it can affect up to 90% of individuals (3). It is characterized by a lack of essential nutrients, which results in a variety of negative outcomes. Malnutrition in the population is caused by multiple factors, including age-related changes in physiology, chronic illnesses, social isolation, financial limitations, and functional limitations (4, 5). Physiological changes in older adults, such as decreased appetite, diminished taste and smell sensations, and reduced gastrointestinal motility, can contribute to poor nutritional intake. Malnutrition can be exacerbated by chronic diseases like cancer, cardiovascular diseases, or diabetes by increasing needs or hindering absorption (6–8). In geriatrics, malnutrition is a significant risk factor due to social isolation and loneliness, as those who live alone may lack adequate social support systems (9). Functional impairments, like mobility limitations and cognitive decline, can impede the ability of an older adult to shop for groceries and prepare meals (10, 11). This frequently leads them to rely on foods that are deficient in nutrients but high in calories. Malnutrition in the older adult can lead to infections, delayed wound healing, functional decline, impaired cognition, and an increased risk of hospitalization and mortality (12, 13). Malnourished older adults are at a higher risk of experiencing prolonged hospital stays and having higher healthcare costs (14). Malnutrition in geriatrics and rehabilitation can hinder the improvement of health outcomes and functional recovery in older adults undergoing rehabilitation (15, 16). Hospitalized older adults undergoing rehabilitation may experience disruptions in their eating habits, and changes in appetite due to medications or medical procedures. Additionally, focusing on therapy could lead to fatigue or increased energy expenditure, which could increase the risk of malnutrition. The recovery process and functional outcomes are at risk due to the significant effects of malnutrition on individuals in rehabilitation. Despite its significance and prevalence, malnutrition frequently remains underdiagnosed and undertreated. Addressing malnutrition is therefore essential to optimize rehabilitation outcomes and improve quality of life for older adults undergoing rehabilitation (17–19). A multidisciplinary approach is crucial in addressing malnutrition in older adult patients undergoing rehabilitation. Various healthcare professionals, such as physicians, nurses, dietitians and occupational and physical therapists, play a vital role in recognizing and managing risk factors related to malnutrition, conducting nutrition assessments, and implementing adequate interventions (20, 21). Nutritional interventions can involve dietary counseling, oral nutritional supplements, modified diets, and feeding assistance for individuals who have swallowing issues. Rehabilitation programs should also focus on enhancing strategies to promote functional independence through activities such as mobility training, providing equipment, and supporting daily living tasks.

Each region is unique due to its cultural heritage, socio-economic status, and geographical factors. The process of identifying malnutrition is influenced by the local environment and the medical expertise involved.

The aim of this study was to assess the prevalence of malnutrition among patients admitted to geriatric rehabilitation in Switzerland. Addressing malnutrition is essential to maximize rehabilitation outcomes and enhance quality of life for older adults undergoing rehabilitation.

## Materials and methods

### Participants

A retrospective analysis of hospital data included 357 patients admitted to the geriatric ward at Lucerne Cantonal Hospital Wolhusen in Switzerland, between 2018 and 2021. The study excluded individuals with mental impairments and those who declined to participate in the study. Participants were divided into four age groups (65–70, 71–80, 81–90 and over 90 years old). They were also divided into three groups based on the Mini Nutritional Assessment (MNA) score: below 17, between 17 and 23.5 and 24 or higher.

### Ethical considerations

The research was conducted in accordance with prevailing international ethical standards and with approval from the Ethics Committee (2020–01051). Additionally, adherence to the ethical principles outlined in the Declaration of Helsinki was rigorously maintained throughout the research process.

### Procedure and materials

Nutritional status was assessed using the standardized Mini Nutritional Assessment Long Form (MNA-LF) questionnaire in accordance with the guidelines of the international professional societies. MNA scores below 17 indicated malnutrition, which required immediate intervention by a nutritionist and the administration of supportive nutritional supplements. Scores between 17 and 23.5 indicated a risk category for malnutrition. Scores of 24 and above indicated a normal nutritional status. The data was collected via the hospital’s electronic database.

### Statistical analysis

The data obtained was analyzed using the computer program for statistical data analysis TIBCO Statistica 13.3. The basic sociodemographic characteristics of the participants were processed by descriptive analysis of response frequencies. The Kruskal-Wallis ANOVA statistical test was used to examine the differences in MNA scores between age groups, while the *post hoc* analysis (LSD) examined the relationships between each groups. Both tests were defined at the level of statistical significance *p* < 0.05. Finally, Pearson’s correlation coefficient was used to compare MNA values and body mass index (BMI).

## Results

The basic sociodemographic characteristics of the participants are listed in Table 1. A total of 357 patients with an average lifespan of 83.6 years was included in the study. The majority were female (65.5%), while men accounted for just over a third of the cases (34.5%). The largest proportion of participants (58%) fell into the 81–90 age group. The majority of respondents (82.1%) were referred to the geriatric department from home, while less than a fifth (17.9%) came from nursing homes. A significant portion of the participants (52.4%) was widowed.

**Table 1:** Basic sociodemographic characteristics of the participants.

Characteristics	*N*	%
Gender		
Women	234	65.5
Men	123	34.5
Age		
61–70	15	4.2
71–80	87	24.4
81–90	207	58
> 90	48	13.4
Average age	83.6	
Habitation		
Home environment	293	82.1
Institution (nursing home)	64	17.9
Marital status		
Married	123	34.4
Divorced	20	5.6
Unmarried	27	7.6
Widowed	187	52.4

*N*, number of participants; %, percentage of participants.

An initial analysis revealed a high prevalence of malnutrition in the geriatric population (as shown in Table 2): 31.1% were classified as malnourished (MNA < 17), while the following 35.8% were at high risk of malnutrition (MNA 17–23.5), totaling 239 patients (66.9%).

**Table 2:** Values of the MNA scores achieved.

MNA score	*N*	%
< 17	111	31.1
17–23.5	128	35.8
≥ 24	118	33.1

MNA, Mini Nutritional Assessment score; *N*, number of participants; %, percentage of participants.

The average Body Mass Index (BMI) was 24.4 (Table 3), categorizing participants into the normal weight range. The average duration of rehabilitation for patients for sample was 20.4 days.

**Table 3:** The average values of BMI and rehabilitation days of the participants.

	Mean	SD
BMI	24.4	4.8
Rehabilitation length/day	20.4	6.8

BMI, body mass index; Mean, average of a data set; SD, standard deviation.

The Kruskal-Wallis ANOVA test shows the statistically significant difference in the MNA scores between the age groups (*p* = 0.035), while the post-hoc analysis shows the greatest difference between the participants from the youngest group (61–70) and the patients over 90 years of age (*p* = 0.017).

**Figure 1:**
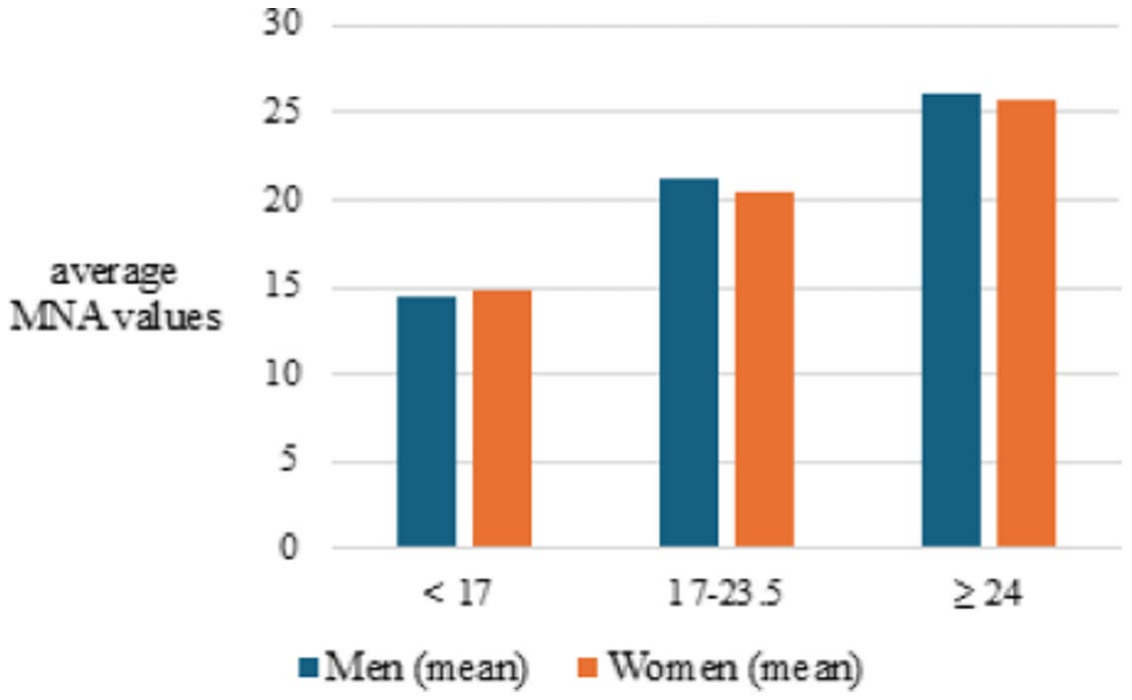
Comparison of MNA score by gender.

On the other hand, the same statistical analysis (K-W ANOVA test, *p* = 0.004) indicates that malnutrition (MNA below 17) was slightly more pronounced in women (Figure 1).

**Figure 2:**
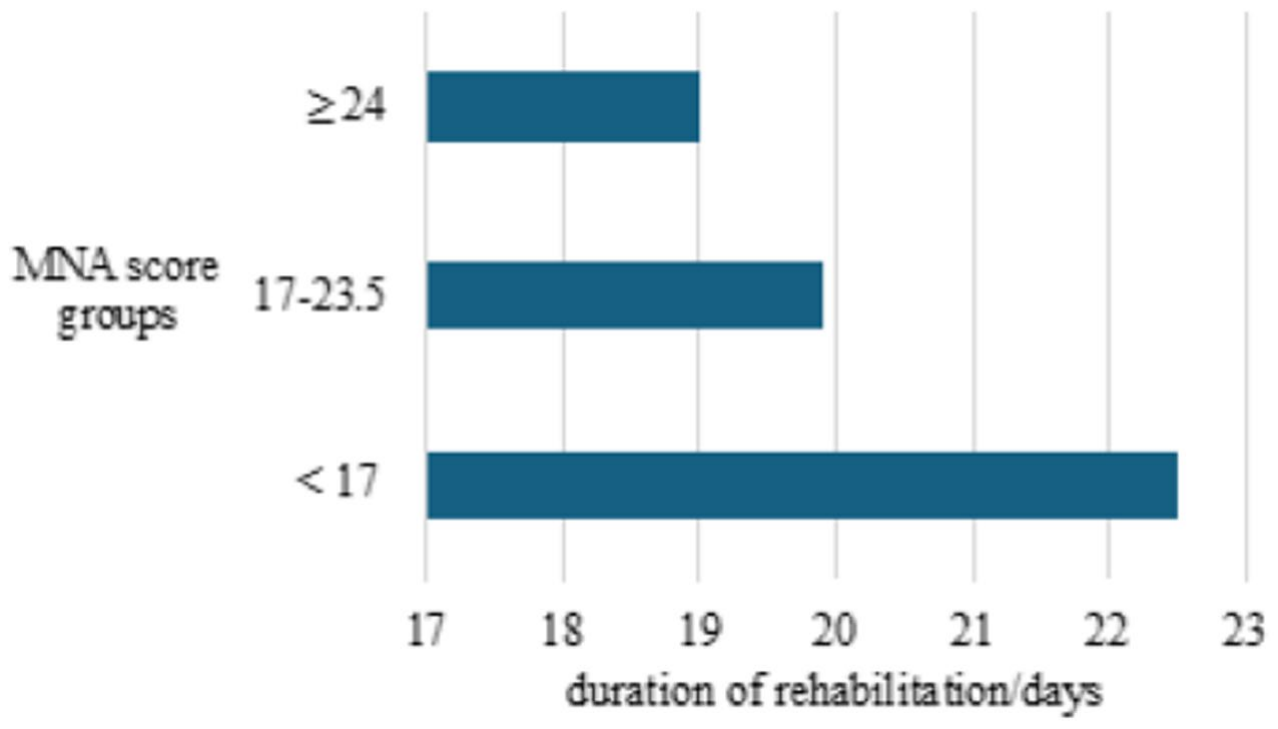
Duration of rehabilitation (expressed in days) in the different MNA score groups.

This was followed by an analysis of the duration of the rehabilitation in days by age group. The duration of rehabilitation ranged from 20.07 ± 6.93 to 22.20 ± 7.50 days, but there was no statistical difference between the observed age groups (*p* = 0.683). However, it is revealed that longer rehabilitation is associated with lower MNA scores (Figure 2).

**Figure 3:**
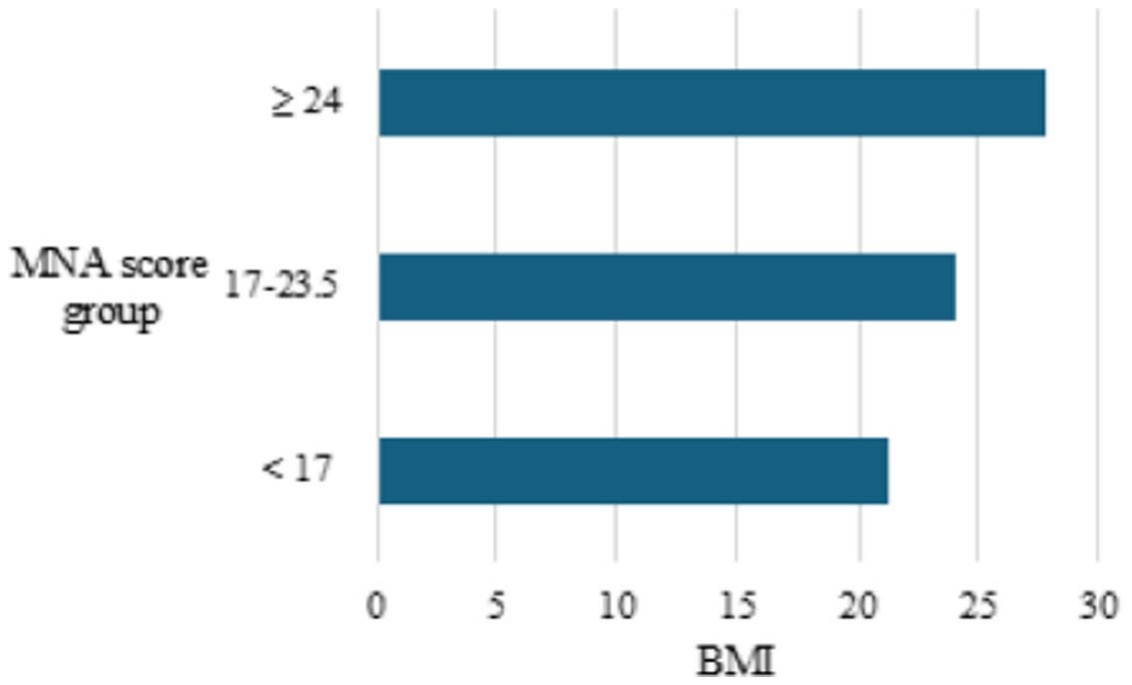
Average BMI values in different MNA score groups.

Considering that malnutrition is often associated with a lower BMI, an analysis of the relationship between MNA values and BMI was performed. The Pearson’s correlation coefficient obtained (*r* = 0.56) indicates a positive correlation. This means that a lower MNA indicates a lower BMI and vice versa, as shown in Figure 3.

## Discussion

Malnutrition is a widespread and concerning issue among the older adult, significantly impacting their health and well-being. This retrospective quantitative study included 357 patients, 224 women and 123 men, admitted to the geriatric ward, with an average age of 83.6 years (Table 1). This study found that 31.1% of participants were undernourished, and an additional 35.8% were at high risk of malnutrition (Table 2). These findings are consistent with previous research, which reports rates ranging from 23 to 46%, with rates as high as 90% among hospitalized older adult individuals (2, 3). Given Switzerland’s high standard of living, this result may seem unexpected. In 2022, Switzerland’s gross domestic product was 818.64 billion dollars, with a *per capita* GDP of 93,259.91 USD, making it one of the world’s top five wealthiest nations (22). However, it’s essential to note that economic status is one aspect influencing malnutrition in this population segment. Social isolation, lack of support for those living alone, and widowhood also play significant roles (23). Most malnourished individuals in the study were referred from home (82.1%), and less than a fifth (17.9%) from nursing homes, with widows comprising a significant proportion (52.4%). This confirms previous research indicating that social isolation and loneliness are substantial risk factors for geriatric malnutrition (24, 25). Older adult individuals living alone may lack social support, leading to decreased motivation for meal preparation and consumption due to loneliness and depression (26).

Analysis showed a significant difference in the risk of malnutrition across age groups, with the youngest group (65–70) having the lowest risk compared to those over 90 years old, indicating an increased risk with age (*p* = 0.017) (Figure 1). The incidence and risk of malnutrition rise with age, possibly because younger people are less socially isolated and more active in social activities, which can reduce the risk of depression (27). Furthermore, aging brings physiological changes such as decreased mobility, reduced appetite, diminished senses of taste and smell, and slowed gastrointestinal motility, all contributing to inadequate nutritional intake and malnutrition (28, 29). With advancing age comes an increase in illnesses and complications well. The average age of participants in this research was 83.6 years. In terms of gender distribution, women were found to be slightly more susceptible to malnutrition (*p* = 0.004), possibly because women generally live longer, often outliving their partners, leading to psychological challenges like depression, which increases the incidence of malnutrition.

Malnutrition has been shown to delay wound healing, extend recovery from acute illnesses, raise the risks and mortality rates associated with noncommunicable and malignant diseases (6, 30). This study found that rehabilitation duration ranged from 20.07 ± 6.93 to 22.20 ± 7.50 days, with no significant difference between age groups (*p* = 0.683). However, longer periods of rehabilitation were associated with lower MNA scores (Figure 2). This indicates that malnutrition significantly delays the progress of rehabilitation in older adult patients, leading to prolonged recovery times and substantial financial burdens, evidenced by studies some studies (17, 18). By addressing malnutrition in geriatrics and rehabilitation through a comprehensive and interdisciplinary approach, healthcare teams can improve the efficiency of rehabilitation programs, improve functional outcomes, and promote the overall well-being of older adults undergoing rehabilitation. Therefore, routine assessment of the nutritional status of patients in geriatric rehabilitation should be considered a standard part of treatment to accelerate rehabilitation, reduce complications, and improve quality of life at home.

Given that malnutrition is often associated with a lower BMI, an analysis of the relationship between MNA values and BMI was performed (31–33). The Pearson correlation coefficient (*r* = 0.56) indicates a positive correlation, meaning a lower MNA score corresponds to a lower BMI and vice versa (Figure 3). The MNA is a tool used to assess the nutritional status of older adults. Despite being subject to numerous studies, controversies, and differing opinions, the MNA questionnaire significantly contributes to identifying individuals whose malnutrition jeopardizes their normal life and recovery and remains a reliable tool in the treatment of the older adult (34–36). The use of the MNA questionnaire is not yet widespread enough, but each new study confirms its usefulness and the necessity for nutritional status assessment to become a standard part of patient care in acute wards and rehabilitation. The assessment of malnutrition must be widely and routinely applied, taking into account specific characteristics of each medical field and geographical region. This can further improve care quality, speed up patient recovery, enhance treatment quality, and reduce healthcare costs.

One limitation of the study concerning the generalizability of the findings is the relatively small sample size. A larger sample size of older individuals would provide results that could be more widely applicable in a broader medical context regarding the prevalence of malnutrition.

## Conclusion

Malnutrition among the older adult is a concerning issue, with significant health and economic implications. This study highlights that a substantial proportion of older adult individuals are either undernourished or at high risk of malnutrition, particularly those living alone or experiencing social isolation. The findings underscore the importance of regular nutritional assessments using tools like the MNA in geriatric care to identify and address malnutrition early. With a link between MNA scores and longer recovery periods incorporating nutritional assessments into standard patient care can improve recovery rates, enhance the quality of life, and reduce healthcare costs. Addressing the multifaceted causes of malnutrition, including social factors and age-related physiological changes, is crucial for improving older adult care and outcomes.

## Data availability statement

The original contributions presented in the study are included in the article/supplementary material, further inquiries can be directed to the corresponding author.

## Ethics statement

The research was conducted in accordance with prevailing international ethical standards and with approval from the Ethics Committee (2020-01051). Additionally, adherence to the ethical principles outlined in the Declaration of Helsinki was rigorously maintained throughout the research process.

## Author contributions

BM: Writing – original draft, Writing – review & editing. YS: Conceptualization, Writing – original draft. GS-K: Writing – review & editing. UC: Supervision, Writing – original draft. LJ: Data curation, Formal analysis, Writing – review & editing. AL: Data curation, Formal analysis, Writing – review & editing. SŠ: Project administration, Writing – original draft.
